# Chemoselective silicification of synthetic peptides and polyamines

**DOI:** 10.3762/bjnano.6.10

**Published:** 2015-01-08

**Authors:** Maryna Abacilar, Fabian Daus, Armin Geyer

**Affiliations:** 1 Faculty of Chemistry, Philipps-Universität Marburg, 35032 Marburg, Germany

**Keywords:** biomineralisation, biosilicification, NMR spectroscopy, polyamines, silaffin

## Abstract

Biosilicification sets the standard for the localized in vitro precipitation of silica at low orthosilicate concentrations in aqueous environment under ambient conditions. Numerous parameters must be controlled for the development of new technologies in designing inventive nanosilica structures, which are able to challenge the biological templates. A long neglected requirement that came into focus in the recent years are the cellular techniques of preventing unintentional lithification of cellular structures since numerous cellular components such as membranes, DNA, and proteins are known to precipitate nanosilica. The diatom metabolism makes use of techniques that restrict silicification to an armor of silica around the cell wall while avoiding the petrifying gaze of Medusa, which turns the whole cell into stone. Step by step, biochemistry unveils the hierarchical interplay of an arsenal of low-molecular weight molecules, proteins, and the cytoskeletal architecture and it becomes clearer why the organisms invest much metabolic effort for an obviously simple chemical reaction like the precipitation of amorphous silica. The discrimination between different soluble components in the silicification process (chemoselective silicification) is not only vitally important for the diatom but poses an interesting challenge for in vitro experiments. Until now, silica precipitation studies were mainly focused on the amount, the morphology, and composition of the precipitate while disregarding a quantitative analysis of the remaining soluble components. Here, we turn the tables and quantify the soluble components by ^1^H NMR in the progress of precipitation and present experiments which quantify the additivity, and potential cooperativity of long chain polyamines (LCPAs) and cationic peptides in the silicification process.

## Introduction

**Figure 1:**
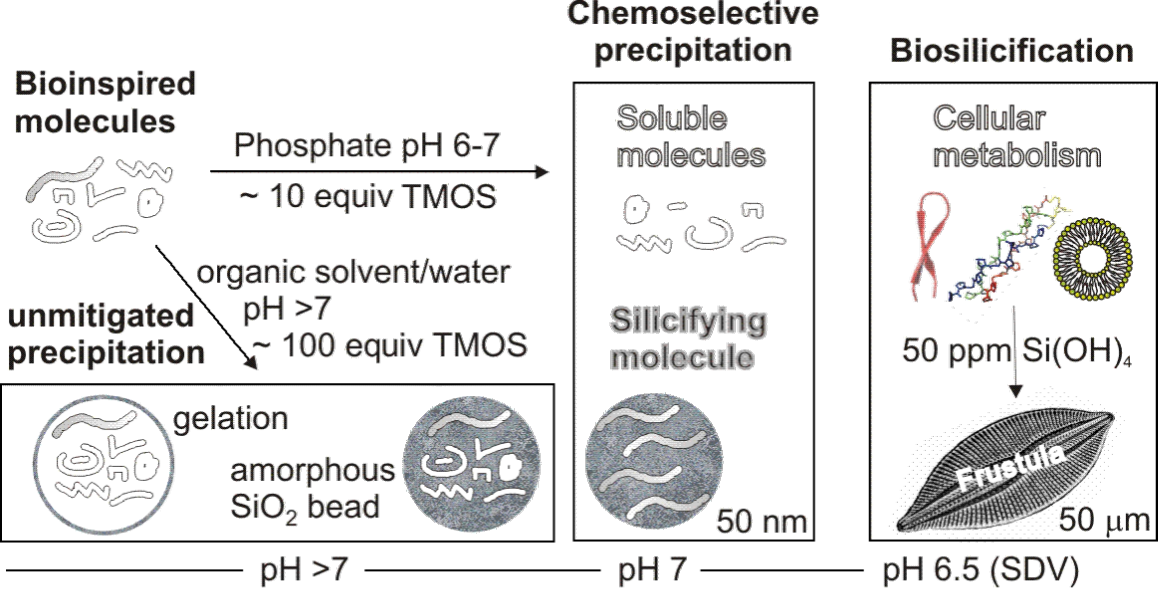
Chemoselective silica precipitation. Different symbols indicate a mixture of organic molecules from which only selected compounds form a precipitate with silicic acid. The remaining solution is analyzed by ^1^H NMR.

Modifications of the Stöber method [1] are in use today for the synthesis of largely monodisperse silica particles with entrapped enzymes for NMR studies [2] or numerous other applications [3]. Generally, one or more molecular species are exposed to orthosilicic acid at pH 7 or higher. Slow or inefficient precipitation is accompanied by gelation of the remainig silicic acid but the primary aim of such experiments is the formation of precipitates with well-defined shapes such as spheres (grey ball in Figure 1) or other morphologies. The unmitigated silicification entraps the dissolved molecules as far as possible in the silica precipitate. Biosilicification however, relies on the sharp differentiation between soluble and entrapped molecules, a sophisticated form of chemoselective silicification. Currently accepted models are the LCPA–phosphate model [4] and the silaffin-matrix hypothesis [5]. Both formulate varying concentrations of soluble components at the surface of the forming silica beads. Poulsen et al. investigated the mutual influence of peptides and LCPAs. Here we investigate the simplest scenario of chemoselective precipitation, which is the differentiation between two dissolved components, a cationic peptide and an oligoamine, that are both capable of precipitating silica on their own (Figure 1). Observing dissolved molecules next to the precipitate gives answers to questions such as these: Is the amine completely consumed by the precipitate formed or is there a fixed N/Si ratio leaving the surplus amine untouched? What happens with the less capable Si precipitator in the presence of the better precipitator? Is there a measurable cooperativity between peptides and amines?

Silica precipitation experiments are time consuming and error-prone because many actions are needed to separate the precipitate of amorphous silica, to dry it, and to weigh it. NMR is no substitute for other analytical methods but ^1^H NMR is a single technology that simultaneously monitors the pH value, viscosity, and amount of dissolved molecules. ^1^H NMR is advantageous for optimizing the experimental settings of silica precipitation process because of the many parameters that are visible in a single spectrum. The consumption of molecules during the precipitation process is quantified as a function of time while constantly monitoring the change in pH from the signal splitting of imidazole and the viscosity of the solution from the half-width of a selected singlet. The greatest benefit lies in the conduction of competition experiments between different types of molecules. By using only a small excess of TMOS, there is no need for stopping the precipitation experiment by addition of HCl after a few minutes. Instead, a molecule of interest can be mixed with a known oligoamine to identify the better precipitator based on the stronger reduction in ^1^H NMR signal intensity.

## Results

### Polyamines and cationic peptides

**Figure 2:**
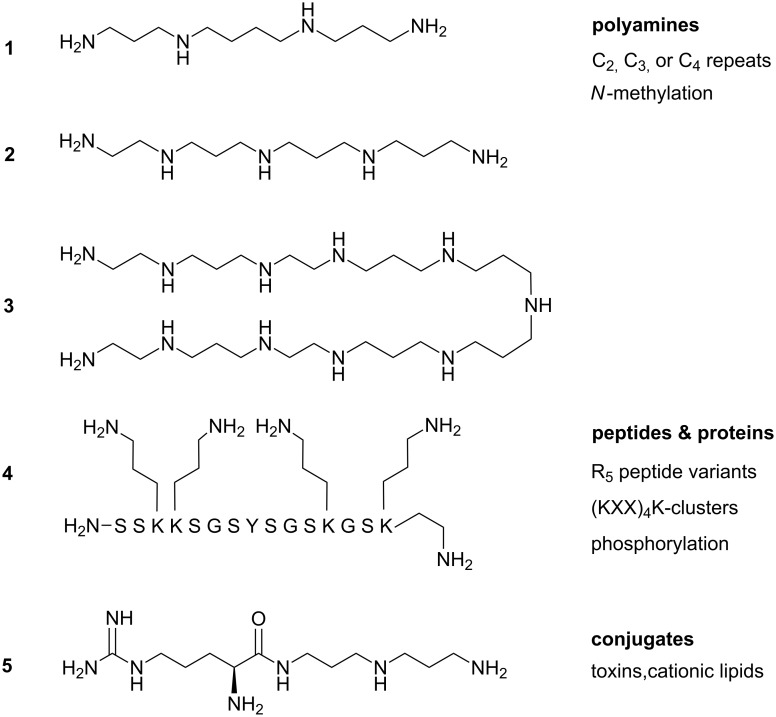
The polyamines **1**, **2**, and **3** have increasing numbers of 4, 5 and 11 basic nitrogens. Peptide **4** is a simplified sequence derived from the silaffins and bears 6 primary amines. Amino acids are characterized by the one-letter code (S Serin, K Lysin, G Glycin, Y Tyrosine). Toxine **5** is the condensation product of Arginine and bis(3-aminopropyl)amine.

The cell wall of diatoms is a composite material with a high content of organic molecules from various compound classes such as oligopropylenamines [6], polycationic peptides [7], proteins [8], and polysaccharides [9]. Even higher contents of organic material are found in sponges in which the biosilica is associated with collagen-type proteins [10]. The common feature of all these organic molecules is their modular assembly. We and others analyzed to what extent the mineralisation process and the morphology of the precipitate depends on the number of propyleneimino repeating units [11], the type of KXXK-boxes in silaffin proteins (K = Lys, X = other amino acid) [12], or the number of POG tripeptide repeating units in collagens [13]. The availability of relatively large amounts of pure material in reproducible quality is a benefit that links organic synthesis to material science. Different from silica-associated molecules of biological origin, which are characterized by structural and compositional microheterogeneity, the chemical synthesis of such molecules yields defined structures and allows for the comparison of individual chain lengths. The distribution of natural oligomers around an average value is replaced with a sequence of individual chain lengths for independent experiments. The aim is to unravel the interplay between different organic compound classes and inorganic components as well as the synergy on different levels of hierarchy from the charge interaction on the atomistic level to the micrometer scale of the frustula structure. Here, we focus on five compounds, which are all either known to or at least expected to precipitate silica (Figure 2). Three amines with increasing number of nitrogen atoms, a basic peptide, and a toxin [14] that is not involved in biomineralization but stands exemplary for other amines capable of silica precipitation.

### Synthetic methods

**Figure 3:**
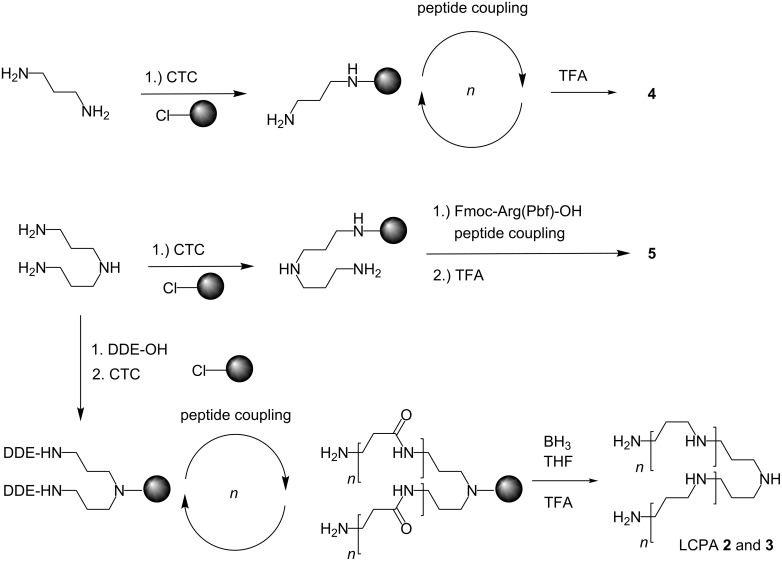
Three synthetic strategies for oligoamines and cationic peptides. The CTC-resin is shown as a grey ball and the arrows represent the coupling and Fmoc deprotection step. The letter *n* stands for the number of coupling and deprotection cycles. DDE = 1-(4,4-dimethyl-2,6-dioxocyclohex-1-ylidene). Top and center: Propylenediamine or bis(3-aminopropyl)amine acts as a spacer for the solid-phase peptide synthesis on CTC-resin. Bottom: Chain extension at both ends of a diamine.

Figure 3 shows the synthetic strategies used to access the molecules **2**–**5**. CTC resin [chloro-(2'-chlorotrityl)polystyrene resin] served as a solid support and was functionalized directly with different amines [14]. The nucleophilicity of one nitrogen of 1,3-propylenediamine was annihilated by tritylation with CTC resin (Figure 3 upper row) while the other peripheral amine remains reactive for peptide coupling. The HBTU/HOBt-mediated condensation of a Fmoc-acylated amino acid and cleavage of the temporary protecting group Fmoc with piperidine was repeated in 15 cycles to obtain CTC-bound precursor of peptide **5** which was finally Boc-deprotected by TFA and simultaneously cleaved from the resin. This peptide bears an additional cationic charge instead of the unproductive C-terminal carboxylate, which would be obtained from traditional solid-phase peptide synthesis. CTC-resin has a double function here because it acts as a protecting group and as a solid support. This strategy can be expanded to other amines such as bis(3-aminopropyl)amine (sometimes called norspermidine) shown in the center of Figure 3. Again only one of the two primary amines reacts with the resin because the secondary amine is sterically too demanding to be tritylated. Avoiding large excess of acylating reagent, toxin **5** was obtained directly in high regioselectivity for acylation without necessity of a N-protecting group on the triamine. Protecting groups on both primary amines lead to a complementary reactivity of bis(3-aminopropyl)amine, now enabling the secondary amine as the only remaining nucleophile to react with the CTC-resin. This is shown in the bottommost synthesis of Figure 3. For the synthesis of **3**, we introduce this handy method, which halves the number of synthetic transformations. Hydrazinolysis cleaved the DDE groups allowing the simultaneous chain extension at both primary amines. Fmoc-β-Ala-OH and Fmoc-Gly-OH were coupled to a suspension of this resin with HBTU. Key step in the synthesis of LCPAs is the borane reduction of oligoamides developed by the groups of Hall [15] and Houghten [16], which we adapted for the synthesis of LCPAs on trityl resin [11]. The amide reduction with excess of the THF-complex of borane removed the amide oxygen to obtain C2 and C3 extensions of the oligoamine. Borane–nitrogen complexes were destroyed in several washing cycles with piperidine before final TFA cleavage of the LCPA from the resin. LCPAs **2** and **3** were obtained with this strategy.

### NMR studies

**Figure 4:**
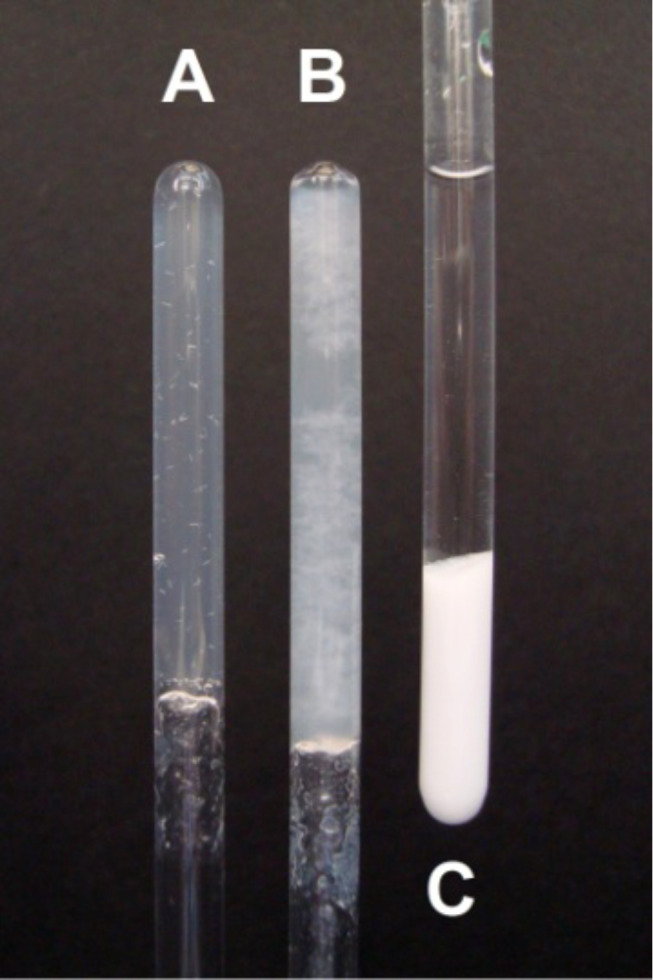
Precipitation of orthosilicic acid with amine **1** in NMR tubes at pH 6.5 (A), 7 (B), and 10 (C). Inefficient silicification in A and B is accompanied by gelation and high viscosity of the reaction mixture. Complete precipitation under the conditions of C shows a sharp separation between the clear solution and the precipitate which is separated by centrifugation to obtain a high-resolution ^1^H NMR spectrum again.

A constant low concentration of orthosilicic acid is expected to be advantageous for chemoselective silicification studies instead of a single addition of a large excess of tetramethyl orthosilicate (TMOS), sometimes exceeding more than 100 equivalents. With the aim of obtaining a constant release of orthosilicic acid from TMOS we initially intended to slow down TMOS hydrolysis by organic solvents. Even the intermediates of TMOS hydrolysis are easily identified by ^1^H NMR in DMSO (Figure S1, Supporting Information File 1) but precipitation studies were not successful because the silica precipitation is slowed down, too. As consequence the unwanted background gelation dominates and the amount of residual water strongly influences the outcome of the experiments. The TMOS hydrolysis is much faster in aqueous environment and all TMOS was available as orthosilicic acid at the beginning of the NMR experiments (Figure S2, Supporting Information File 1). All measurements were conducted in buffered solution of deuterated water to simplify the experimental setting of the NMR measurement. Instead of a single addition of TMOS, we added small amounts (less than ten equivalents) stepwise until all organic molecules were precipitated and the integral in the ^1^H NMR approached zero. In the precipitation studies of isolated molecular species it made no difference whether TMOS was added in a single step or in several portions. Orthosilicic acid was consumed for silica precipitation as long as a molecule that is capable of silica precipitation remains in solution. Whether TMOS was added in a single step or in portions had no influence on the overall result. For the precipitation studies of more than one dissolved component it was possible to measure the ratio of the dissolved molecules after each stepwise addition of TMOS as described in the competition experiments below. The dynamic range of modern NMR spectrometers (16 bit digitizer) is big enough to resolve the signal intensity of the organic molecule in the presence of a large methanol signal from the TMOS hydrolysis. Their relative intensities quantify the excess of orthosilicic acid present in the silicification experiment. Three typical outcomes of the silicification experiments are shown in Figure 4 in which gelation is directly visible from the clouding and solidification of the solvent (experiments A and B). Only precipitation of type “C” yields amorphous silica from the complete precipitation of orthosilicic acid without gelation of any remaining dissolved silica.

**Figure 5:**
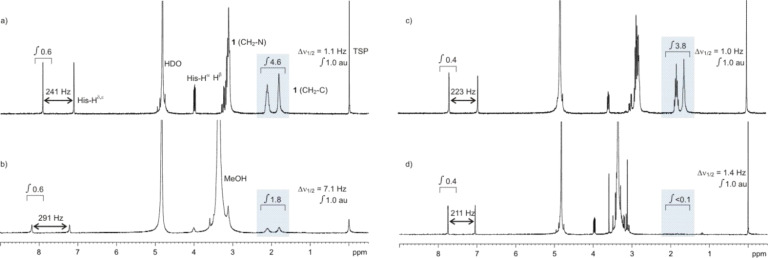
^1^H NMR spectra (300 MHz, 300 K) of **1** a) without and b) in the presence of orthosilicic acid at pH 6 and the same experimental setting at pH 11 c) and d). TSP serves as an internal standard and the signal intensity (integral) was set to 1 a.u. (arbitrary units). Histidine is present as a second internal standard because the splitting of the imidazole CH groups (His-H^δ^ and His-H^ε^) depends on the pH value in the region around the p*K*_a_ value of the imidazole side chain. Signals of **1** which are not influenced by the resonance of the released methanol from the TMOS hydrolysis are highlighted by a blue box. Further details are given in the text.

The increase in macroscopic viscosity, which is directly visible in the upside-down turned tubes in Figure 4, corresponds to the microscopic viscosity, which is visible as line broadening of all signals but quantified as the half width of the trimethylsilyl propanoic acid (TSP) singlet that serves as an internal standard. Gelation is accompanied by a significant increase of line broadening. While the initial viscosities of the buffered solutions are characterized by values which do not surpass 1.5 Hz, a factor of 10 is typical for the gelated NMR tubes. A second internal standard is histidine which has a p*K*_a_ value of around 6.5 [17]. The signal separation of the imidazole singlets of histidine show significant changes around this pH value although it does not interfere with the precipitation process. In spite of its three nitrogen atoms, histidine does not get incorporated into the silica, which shows the special properties of the other investigated oligoamines. Precipitation studies were performed with all compounds shown in Figure 1. Typical NMR spectra under different pH conditions are shown in Figure 5 for compound **1**. Two well separated methylene groups of **1** at 2 ppm, which are not influenced by the released methanol from the added TMOS, are highlighted with a blue box. The increase of the line broadening at pH 6.5 (Figure 5a and Figure 5b) affects all signals but the signal integrals do not change except for **1,** which loses half of its intensity. Short-chained amines are not qualified to precipitate silica at this pH and therefore gets incorporated only by 50% while the remaining orthosilicic acid forms a gel. A further decrease in pH is detected by the increase of the signal splitting of the imidazole from 241 Hz (a) to 291 Hz (b). At significantly higher pH (Figure 5c,d) under typical Stöber conditions all amines are qualified to precipitate silica while the buffer keeps the high pH value. Therefore the signal of **1** is completely absent in d) and the signal splitting of imidazole does not change significantly. Furthermore, a change in solvent viscosity is not detectable. **1** becomes completely incorporated into the silica under strongly basic conditions and a low Si/N ratio of 4 is calculated under the assumption that the orthosilicic acid is consumed completely.

These NMR experiments were conducted for all compounds **1**–**5** (Table 1). With compound **1** silica cannot be precipitated at pH values below 7 without gelation [18]. All other experiments in Table 1 were conducted under high resolution conditions without gelation and polyamine **3** is quantitatively precipitated even at the lowest investigated value of pH 5.5. An amount of 50 equivalents of TMOS is precipitated by both longer LCPAs **2** and **3**, respectively, in phosphate buffer at slightly acidic pH values. Si/N is the ratio between orthosilicic acid and the number of basic nitrogens in the investigated compound minus the remaining peptide. Peptide **4** precipitates orthosilicic acid effectively but gets much less incorporated with a Si/N ratio above 100. Three additions of 40 equivalents of TMOS are necessary to precipitate it completely from solution. Toxin **5**, although completely unrelated to biosilicification, is able to precipitate silica, too. The dissolved molecules influence the buffer capacity and small changes in pH can influence the precipitation behavior of two different molecules. The reproducibility of each experimental setting was excellent but we consider it difficult to compare precipitation experiments of different molecules. Therefore, we designed experiments in which one amine serves as an internal standard for precipitation and the precipitation capacity of the second component can be easily judged by measuring a ^1^H NMR spectrum of the remaining solution after precipitation. The better precipitator is incorporated in the solid silica and removed from solution while the other component remains and yields signal intensity in the NMR spectrum. The technique can be expanded to more than two soluble components as long as at least one signal intensity is separated on the chemical shift scale. In this study, LCPA **3** is the compound that precipitates silica most efficiently and it is probably the most promising one to precipitate in the presence of other dissolved organic molecules. For **1** we observe significant chemoselectivity at neutral pH or slightly below. Commercially available spermine (**1**) was chosen as a reference to make the experimental setting independent from molecules which are only available in our group.

**Table 1:** Silica precipitation experiments. The final column is the initial splitting of the imidazole signals which correlates with the observed pH value in brackets. The lower three rows list the numerical values for the bar diagrams in Figure 6.

entry	compound	Si/N	random error^a^	equivalents TMOS, % peptide	Δν_1/2_ [Hz]	Δδ H^δ^–H^ε^, (pH)

1	**1**	33:1	±0.3	16, 39% (gelation)	1.1, 7	241, (6)
2	**1**	4:1	±0.45	16, 0%	1.0, 1.4	223, (11)
3	**2**	50:1	±0.25	50, 0%	1.1, 1.1	234, (6.8)
4	**3**	50:1	±0.25	50, 0%	1.1, 2.2	249, (5.5)
5	**4**	142:1	±0.34	40, 81%; 40, 34%; 40, 0%	0.9, 1.3	235, (6.8)
6	**5**	98:1	±0.55	40, 32%; 40, 0%	0.9, 1.2	230, (7)
7	**1 + 4** (1:1)^b^	80:1	±0.55	40, 30% (**1**), 96% (**4**); 40, 0%	0.9 1.6	231, (7)
8	**1 + 4** (2:1)	72:1	±0.35	28, 72% (**1**), >98% (**4**); 28, 0% (**1**), 77% (**4**)	1.0 1.1	248, (6)
9	**1 + 5** (1:1)	24:1	±0.85	7, 39% (**1**), 74% (**5**); 7, 28% (**1**), 66% (**5**); 14, 15% (**1**), 35% (**5**); 30, 0% (**1**), 0% (**5**)	1.0 1.0	224, (7)

^a^The random error depends on the signal-to-noise ratio and uncertainties of signal integration in each ^1^H NMR spectrum. The experimental error increases parallel with the addition of TMOS because of the decreasing signal-to-noise ratio for the molecules of interest in the spectra. No error is given when the remaining amine is below the detection limit. Random errors are given only for the first addition of TMOS. ^b^Ratio in brackets.

**Figure 6:**
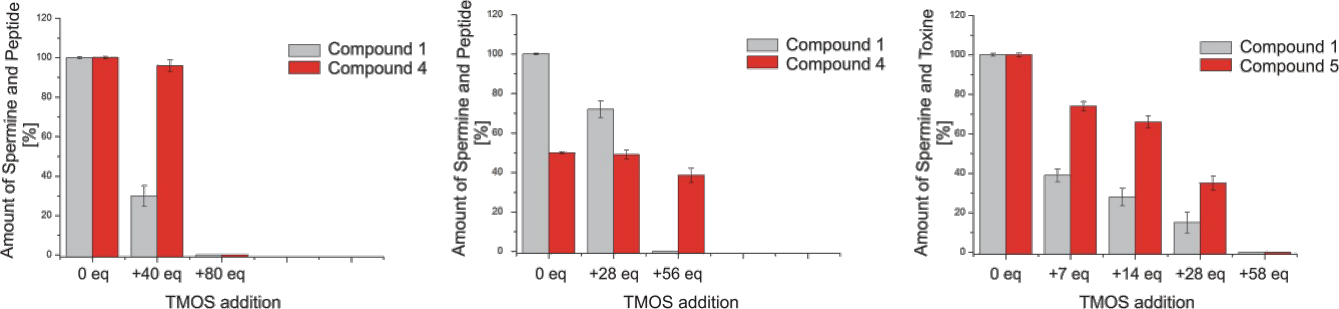
Silica precipitation competition experiments. Experimental details are according to the single component precipitations in Table 1. The addition of TMOS is shown on the *x*-coordinate and the starting conditions are set to 100% for better comparison. The left diagram shows spermine (**1**) vs R_5_ peptide **4** in a 1:1 ratio (10 μmol/mL each) and the middle diagram shows them in a 2:1 ratio (15 μmol/mL both). In both precipitation experiments, **1** is consumed before a relevant amount of **4** precipitates. Right: **1** vs **5** in a 1:1 ratio (5 μmol/mL each). Again, **1** is the better precipitator although both organic molecules are consumed during the mineralization process.

Figure 6 shows competition experiments with two amines competing for the silicic acid. From an equimolar mixture of **1** and **4**, two thirds of the amine precipitate without affecting the peptide concentration. Addition of another 40 equivalents of TMOS precipitates both molecules. Even from the 2:1 ratio of **1** and **4** with 28 equivalents of TMOS, approximately one third of the amine precipitates first. The second addition of the same amount of TMOS eliminates the signals of the amine completely and reduces the amount of peptide to 77% of the starting concentration. The chemoselectivity is less pronounced for the mixture of **1** and **5**, which was titrated with the smallest first addition of only 7 equivalents of TMOS. From these competition experiments can be deduced that **5** is a better precipitator than **4**. An interesting observation is that **1** is a better precipitator in the presence of peptide **4** at pH 6 (Figure 6 right) than without (Table 1, entry 1).

**Figure 7:**
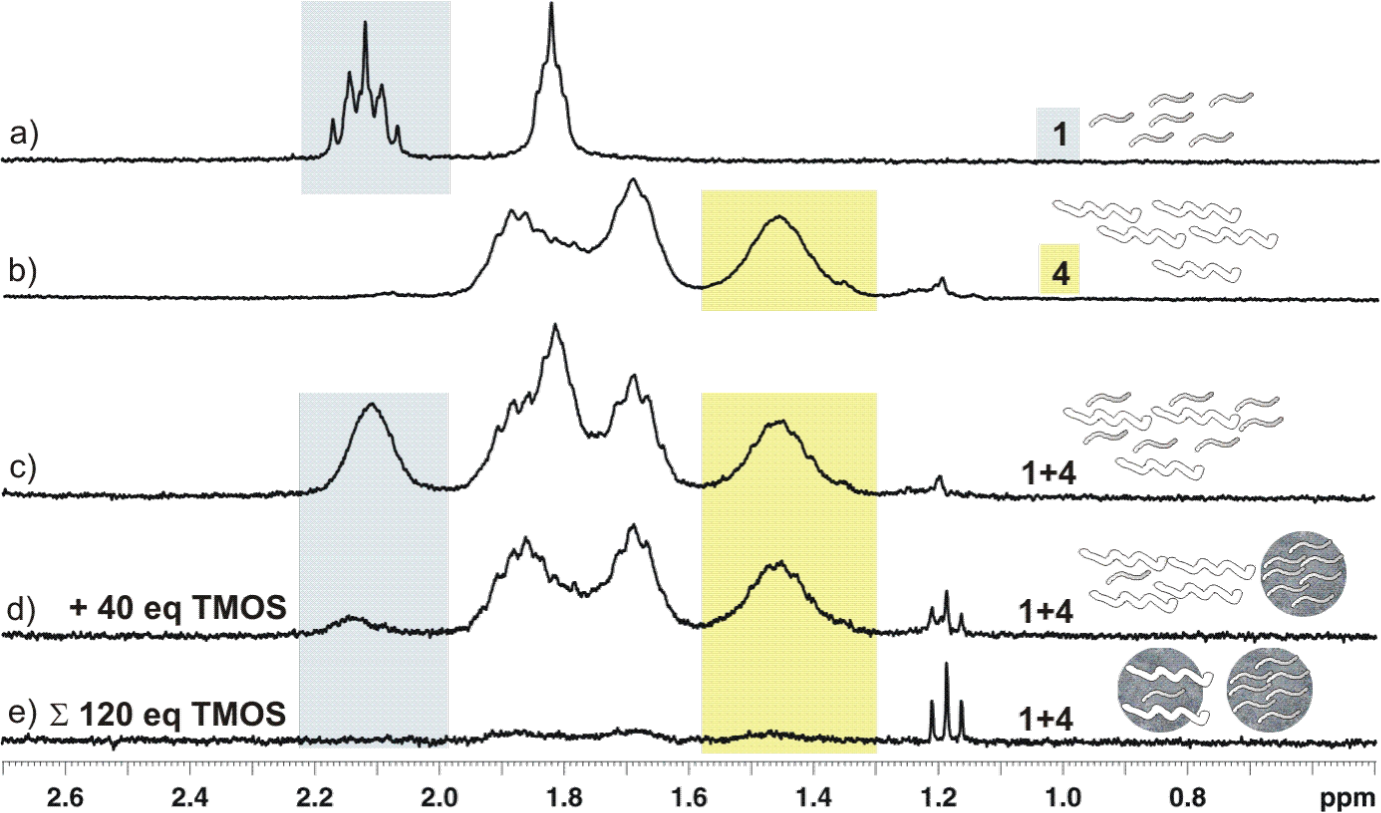
Expansions from the ^1^H NMR spectra (300 MHz, 300 K) of **1** a) and **4** b) and the spectrum of an equimolar mixture of both c) before the addition of orthosilicic acid at pH 7 together with the spectra of the stepwise addition of TMOS in d) and e). A methylene group that is only present in **1** and another that is only present in **4** are highlighted and document the stepwise precipitation of the two molecules. (Triplet at 1.19 ppm EtOH from residual tetraethyl orthoslicate (TEOS) in TMOS.) The complete ^1^H NMR spectra are shown in the Figure S3, Supporting Information File 1).

Exemplary for the experiments of Table 1, the ^1^H NMR spectra of the equimolar competition experiment between amine **1** and peptide **4** (Table 1, entry 7) are shown in Figure 7. The amine and the cationic peptide are not expected to interact under the experimental conditions and the ^1^H NMR of the mixture represents the sum of the two single spectra. The chemoselective silica precipitation is documented in spectrum d) after the first addition of TMOS. The blue methylene group is incorporated in the silica precipitate while the yellow methylene group is still there. Spectrum e) proves that both molecules are competent to precipitate silica under the experimental conditions while the internal standards histidine and TSP remain in solution in all spectra.

## Discussion

LCPA phosphate microdrops are competent to precipitate silica [19] and the silaffin-matrix model attempts to explain silica precipitation inside of the silica deposition vesicle (SDV) at pH values as low as 5.5. Under these conditions, the nanostructure forms a template for the localized silicon dioxide precipitation in LCPA-rich but silaffin-poor areas [20]. From the numerous physical and experimental parameters, which differ from the literature experiments, only the chemical parameter of micro-heterogeneity is resolved in our experiments, which consist of two-component mixtures of defined concentration of each individual molecular species. Polyamines and peptides compete for silicic acid in the NMR tube. Further parameters such as the excess of silicic acid, the pH value, and phosphate concentration are selected by us based on former precipitation experiments. In competition experiments of peptide **4** (Table 1, entry 7) and amine **5** (Table 1, entry 9), amine **1** serves as an internal standard that identifies **5** as the better precipitator than **4**, without the necessity of conducting the competitive precipitation of **4** and **5**.

## Conclusion

In conclusion, it is not our intension to advertise for NMR as a substitute for classical silica precipitation experiments but as a fast, rich in information, and fail-proof additional method for the identification of in vitro conditions for the development of synthetic silica nanocomposites. The better organic template is entrapped in the inorganic precipitate while the others remain in solution. This straightforward method identifies cooperativity of bioorganic templates in solution and can be easily transferred to other mineralization experiments.

## Experimental

The NMR spectra were recorded at 300 MHz and the pH values are not corrected to pD. Synthesis details are given in Supporting Information File 1.

## Supporting Information

File 1 Synthesis details.

## References

[1] Stöber W, Fink A, Bohn E (1968). J Colloid Interface Sci.

[2] Guisan J M (2006). Immobilization of Enzymes and Cells.

[3] Patwardhan S V (2011). Chem Commun.

[4] Kröger N, Deutzmann R, Bergsdorf C, Sumper M (2000). Proc Natl Acad Sci U S A.

[5] Poulsen N, Sumper M, Kröger N (2003). Proc Natl Acad Sci U S A.

[6] Sumper M, Lehmann G (2006). ChemBioChem.

[7] Kröger N, Deutzmann R, Sumper M (1999). Science.

[8] Richthammer P, Börmel M, Brunner E, van Pée K-H (2011). ChemBioChem.

[9] Ogasawara W, Shenton W, Davis S A, Mann S (2000). Chem Mater.

[10] Ehrlich H, Deutzmann R, Brunner E, Cappellini E, Koon H, Solazzo C, Yang Y, Ashford D, Thomas-Oates J, Lubeck M (2010). Nat Chem.

[11] Bernecker A, Wieneke R, Riedel R, Seibt M, Geyer A, Steinem C (2010). J Am Chem Soc.

[12] Wieneke R, Bernecker A, Riedel R, Sumper M, Steinem C, Geyer A (2011). Org Biomol Chem.

[13] Weiher F, Schatz M, Steinem C, Geyer A (2013). Biomacromolecules.

[14] Nash I A, Bycroft B W, Chan W C (1996). Tetrahedron Lett.

[15] Hall D G, Laplante C, Manku S, Nagendran J (1999). J Org Chem.

[16] Nefzi A, Ostresh J M, Houghten R A (1999). Tetrahedron.

[17] Silverstein T P (2012). J Chem Educ.

[18] Belton D J, Patwardhan S V, Annenkov V V, Danilovtseva E N, Perry C C (2008). Proc Natl Acad Sci U S A.

[19] Sumper M, Brunner E (2006). Adv Funct Mater.

[20] Sumper M, Kröger N (2004). J Mater Chem.

