# Supplementary material for: Chemoselective silicification of synthetic peptides and polyamines
Source: Beilstein J Nanotechnol. 2015 Jan 8;6:103–10. doi: 10.3762/bjnano.6.10 (PMC4311759; doi:10.3762/bjnano.6.10)
Supplement: File 1 — Synthesis details. [file Beilstein_J_Nanotechnol-06-103-s001.pdf]

Supporting Information

for

Chemoselective silicification of synthetic peptides and polyamines

Maryna Abacilar, Fabian Daus and Armin Geyer*

Adress: Faculty of Chemistry, Philipps-Universität Marburg, 35032 Marburg, Germany

Email: Armin Geyer* - geyer@staff.uni-marburg.de

* Corresponding author

Synthesis details

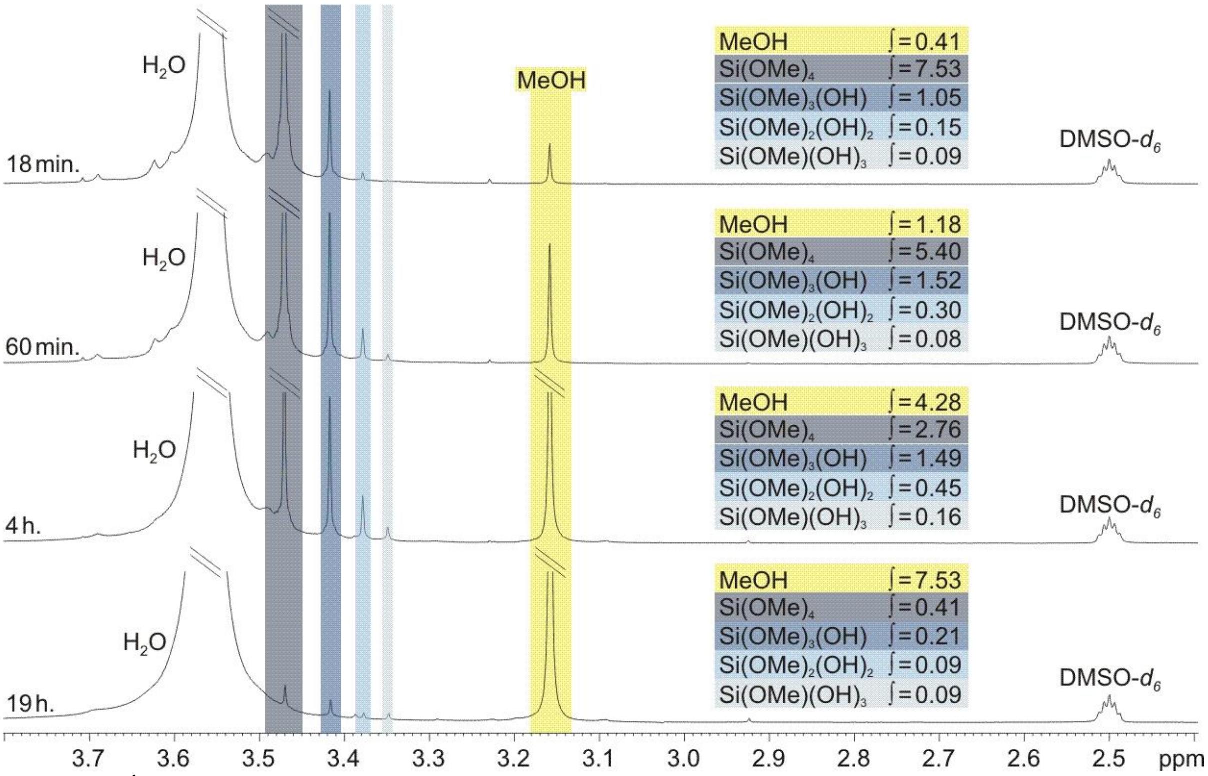

Figure S1: ^{1}H NMR spectra (300 MHz, 300 K) of the TMOS hydrolysis in DMSO-*d*6. Each integral quantifies the amount of one of the transiently observable mono-, di-, or triesters.

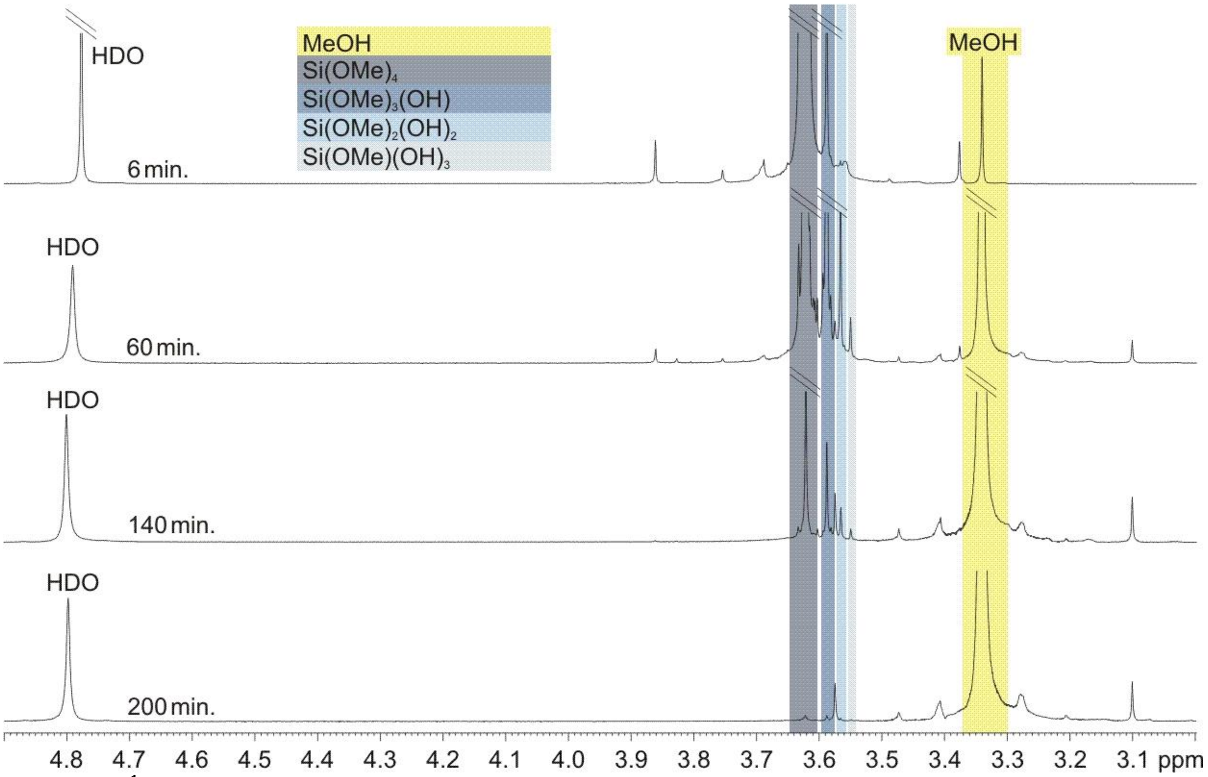

Figure S2: ^{1}H NMR spectra (300 MHz, 300 K) of the TMOS hydrolysis in D_2O. No integrals are quantified for the transiently observable mono-, di-, or triesters. The hydrolysis is complete in phosphate buffer after 15 min.

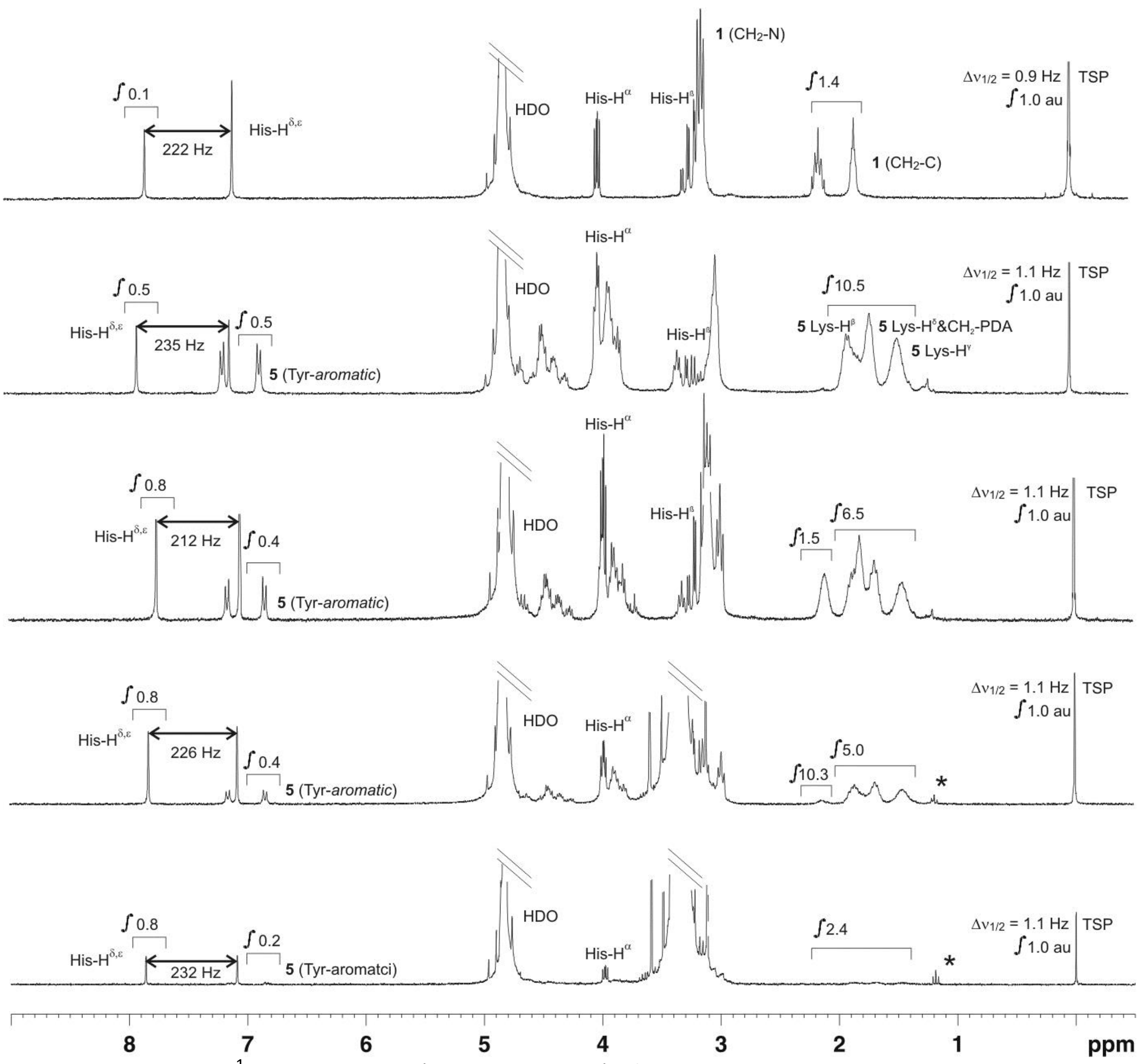

Figure S3: Complete ^{1}H NMR spectra (300 MHz, 300 K) of Figure 6.

Synthetic methods

Synthesis of toxin 5: Norspermidine was added to a suspension of the CTC-resin (1.0 g, 1.8 mmol/g) (3.5 g, 27 mmol) in anhydrous DMF at room temperature under nitrogen atmosphere. After shaking for 2 min, 25% MeOH in DCM (vol %) was added. After 1 h the resin was filtered, washed with 25% Et_3N in DMF (vol %), DMF, MeOH, DCM, and then dried in vacuo for 8 h. The loading of the resin was determined gravimetrically. Fmoc-L-Arg(Pbf)-OH (0.8 g, 0.4 mmol), HBTU (0.2 g 0.4 mmol), HOBt (0.05 g, 0.5 mmol) and DIPEA (102 µL, 0.6 mmol) were added to a suspension of the above resin (0.3 g, 0,65 mmol/g) in DMF at r.t. under nitrogen atmosphere. After shaking for 1 h, the resin was filtered, washed with DMF, MeOH, DCM and dried in vacuum for 2 h. The Fmoc group was removed by treatment with 25% piperidine in DMF (vol %) for 30 min (1 × 10min, 1 × 20 min). To a mixture of the resulting resin TFA/H2O/PhOH/TIPS (88/5/5/2) (% v/v/w/v) were added. After shaking for 4 h, the resin was filtered and the filtrate precipitated from dry ether. The precursor-toxin (sFTX-3.3) was lyophilized after several washing steps in ether.

Synthetic procedure for polyamines: Double-protected spermine was prepared from spermine (1.1 mL, 7.2mmol) in a reaction with 2-acetyl dimedone (2.6 g, 14.4 mmol) by refluxing in ethanol for 4 h. To a suspension of the CTC-resin (1.0 g, 1.8 mmol) in DCM (5 mL) double-protected spermine (3.5 g 7.6 mmol) in presence of Et_3N (0.2 g, 2.2 mmol) was added at r.t. After shaking for 8 h, the resin was filtered, washed with DMF, MeOH, DCM. Afterwards the unreacted 2-chlorotrityl groups were quenched with methanol for 1 h. The loading was determined gravimetrically. Then Dde-protected primary amines were selectively deprotected with 2% hydrazine in DMF (vol %). Free amines were acylated by using fourfold excess of the appropriate acid, activated by HBTU (2 equiv), HOBt (2 equiv) and DIPEA (3 equiv) for each active site in DMF. The Fmoc group was removed by treatment with 25% piperidine in DMF for 30 min.

General procedure for borane reduction: 1 M solution of borane in THF (25 equiv per amide) was added to the dry resin under nitrogen atmosphere in a flame-dried flask. After 15 min at room temperature, the reaction mixture was heated to 55 °C and kept at this temperature for two to five days depending on the length of the polyamines. After washing with THF and MeOH, the work-up under alkaline conditions follows. Therefore the resin was treated three times with piperidine (25 mL/g resin) at 55 °C for 2 × 2 h and 1 × 12 h. Finally, the resin was suspended in 2% hydrazine in DMF (10 mL/g resin). After shaking for 30 min the resin was filtered, washed with DMF, MeOH, and DCM, the resin was dried for 8 h under high vacuum. Cleavage of the oligoamines from the support was accomplished by a cleavage procedure by using 95% aqueous TFA. Excessive TFA was removed in vacuum and precipitated with diethyl ether. The white powder was dried under high vacuum.

General procedure for Fmoc solid phase peptide synthesis: For the coupling of the amine on the support, the resin was first swollen in DMF for 30 min at r.t under nitrogen atmosphere. To a suspension of the resin Fmoc-protected amino acid (2 equiv), HBTU (2 equiv), HOBt (2 equiv) and DIPEA (3 equiv) was added in the given order. The reaction mixture was shaken for 1 h. Then the resin was filtered, washed with DMF, MeOH, DCM. Fmoc-deprotection was achieved with 25% piperidine in DMF (1 × 10 min, 1 × 20 min) followed by the previously mentioned washing steps. To cleave the peptide from the resin TFA/TIPS/H2O (95/2.5/2.5) were added to the resin for 2 h at r.t. The combined filtrates were concentrated under high vacuum and precipitated from diethyl ether.

Statistical analyses: Random and systematic errors appearing herein are due to the experiment**.** In the values of both pipetting the standard buffer solution, and the solution in this model substances are included, as well as weighing error smaller amounts of both. The derived and discussed ratios here or their dynamics have been detected or monitored by highly sensitive ^{1}H NMR experiment. The random errors were not visible in the 1H NMR and therefore neglected. Thus, the results obtained are essentially reading errors influenced by the random that come from the determination of the integrals. Since all experiments have been measured on a single instrument, relaxation-induced systematic error of ±5% was not taken into account. However, taken into consideration the personal random error, the signals integrate 1H NMR. This was estimated to be ±1%.

Graphical representations were performed with Origin software version 8.5.
